# Antiplatelet Resistance in Coronary Artery Bypass Grafting: A Systematic Review

**DOI:** 10.1155/2024/1807241

**Published:** 2024-06-15

**Authors:** Myat Soe Thet, Amir Khosravi, Samson Egbulonu, Aung Ye Oo

**Affiliations:** ^1^Department of Surgery and Cancer, Imperial College London, London, UK; ^2^Department of Cardiothoracic Surgery, Liverpool Heart and Chest Hospital, Liverpool, UK; ^3^Department of Cardiothoracic Surgery, St Bartholomew's Hospital, London, UK

## Abstract

**Background:**

This systematic review examines the occurrence and implications of resistance to primary antiplatelet agents, aspirin and clopidogrel, often utilised in patients undergoing coronary artery bypass grafting (CABG), alongside the methodologies for assessment of such resistance.

**Methods:**

An extensive literature search across various databases such as PubMed, MEDLINE via Ovid, Embase, and Cochrane CENTRAL until May 2024 was conducted to identify studies evaluating antiplatelet resistance in on-pump and off-pump CABG patients. Following quality assessment, only high-quality studies were incorporated into this review.

**Results:**

This review included 19 studies with 3,915 patients, four of which were randomised controlled trials and 15 were observational studies. Aspirin resistance incidence ranged from 11.0% to 51.5%, while clopidogrel resistance was 22%. Antiplatelet resistance, assessed through a wide variety of methods, was associated with a 13 times increase in the risk of vein graft occlusion and increased rates of mortality, myocardial infarction, and target vessel revascularisation in the case of clopidogrel resistance. The effect of cardiopulmonary bypass on antiplatelet resistance remains ambiguous.

**Conclusion:**

The academic literature lacks a standardised definition for antiplatelet resistance. Assessment methodologies greatly vary, leading to noninterchangeable outcomes. While aspirin resistance has a conflicting overall significant impact on adverse outcomes, clopidogrel resistance correlates with poorer clinical outcomes.

## 1. Introduction

In patients undergoing coronary artery bypass grafting (CABG), the two commonly used antiplatelet agents are aspirin and clopidogrel. Early aspirin within 48 hours after CABG serves to mitigate mortality risk and the incidence of organ ischemia in the brain, kidneys, heart, and gastrointestinal tract [1]. The initiation of aspirin soon after CABG surgery has also been substantiated to significantly enhance the patency of vein grafts without increasing the bleeding risk [2]. Aspirin functions by irreversibly acetylating the platelet cyclooxygenase (COX) enzyme, hence inhibiting the conversion of arachidonic acid to thromboxane A2 (TxA2). Due to its inherent chemical instability, TxA2 undergoes conversion into the stable, inactive thromboxane B2 (TxB2). The resultant metabolite is 11-dehydro-TxB2, and both are detectable in urine.

Dual antiplatelet therapy, incorporating aspirin and clopidogrel, mitigates the incidence of thrombotic complications following acute coronary syndrome (ACS) [3]. Clopidogrel is an adenosine diphosphate (ADP) receptor antagonist. It inhibits platelet activation by binding to the P2Y12 receptor irreversibly. This dual therapy leads to a reduction in all-cause mortality and improves vein graft patency, exerting more significant effects on ACS patients undergoing CABG surgery [4, 5].

However, antiplatelet resistance noted in a subset of patients has been implicated in early graft failure, attributed to suboptimal responsiveness to the antiplatelet agents administered [6]. Though a range of tests exists for assessing antiplatelet resistance, their precision varies, and correlations among them are not consistent [7]. This systematic review intends to explore contemporary practices, the application of assessment methodologies, and the ramifications of antiplatelet resistance in patients undergoing CABG surgery.

## 2. Methods

The present systematic review was conducted in accordance with the Preferred Reporting Items for Systematic Reviews and Meta-Analyses (PRISMA) guidelines [8]. Ethical approval or patient consent was not sought as this review solely relied on preexisting published studies.

### 2.1. Search Strategy

A systematic search was carried out across major databases of PubMed, MEDLINE via Ovid, Embase, and the Cochrane Library Database until May 2024 to identify eligible studies using Boolean operators to achieve maximum sensitivity. The search terms used are “((CABG) OR (Coronary artery bypass graft^*∗*^) OR (Cardiac surgery)) AND ((Antiplatelet) OR (Aspirin) OR (Clopidogrel) OR (Ticagrelor) OR (Antithrombotic)) AND ((Mortality) OR (Morbidity) OR (Graft patency) OR (Survival)) AND ((Resistance) OR (platelet mapping)) NOT (stent).” Bibliographies of relevant studies were also screened manually to identify additional suitable studies.

### 2.2. Study Selection and Data Extraction

The inclusion criteria include human studies, where patients underwent CABG surgery and had received a minimum of one antiplatelet agent during the perioperative phase. The included studies must also provide at least one outcome related to antiplatelet resistance, such as vein graft failure, mortality, or morbidity. Animal studies, case reports, case series, reviews, and non-English articles were excluded.

Two authors independently conducted the database search (MST and SE), reviewed articles for potential relevance, extracted data, and assessed the quality and risk of bias in the included studies. Discrepancies were reconciled through consensus or, if needed, consultation with the third author (AK).

### 2.3. Quality Assessment of Included Studies

The quality of the observational cohort studies was evaluated using the Newcastle-Ottawa Scale (NOS), designating scores above six as indicative of high-quality studies [9]. For the assessment of randomised controlled trials, the Cochrane risk of bias assessment tool was used [10].

## 3. Results

### 3.1. Characteristics of the Studies

**Figure 1:**
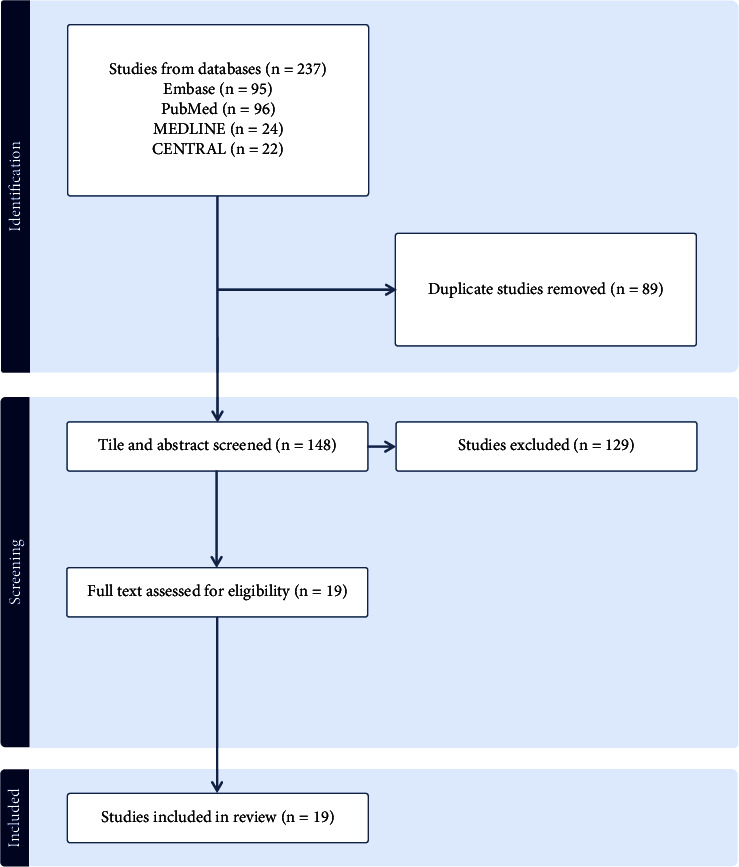
PRISMA flow diagram for study search and selection.

**Figure 2:**
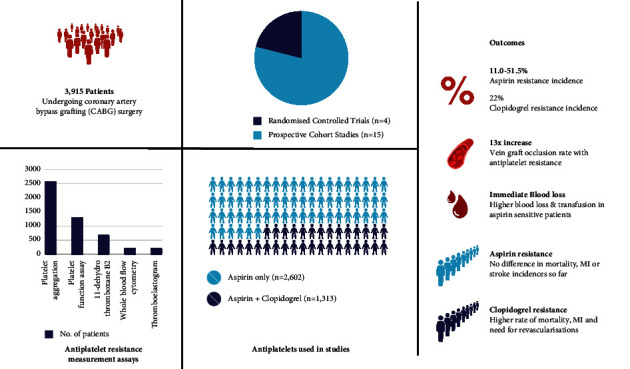
Graphical summary of the included studies.

The systematic search identified 237 studies in total as described in the PRISMA flowchart (Figure 1). After removing 89 duplicates, titles and abstracts of the remaining 148 studies were screened. A further 129 studies were excluded leaving 19 studies for full-text review, after which all 19 studies were found to meet inclusion criteria. Therefore, a total of four randomised controlled trials and 15 observational cohort studies with a total of 3,915 patients were included in the systematic review [11–28]. The specific characteristics of these studies are outlined in Table 1. Each of the 15 observational studies achieved a score of 6 on the Newcastle-Ottawa Scale (NOS), thus affirming them as good-quality observational studies (Supplementary Table 1). Similarly, the four randomised controlled trials were deemed low-risk and qualified as high-quality studies (Supplementary Figure 1) [10]. The graphical summary is illustrated in Figure 2.

### 3.2. Assessment of Antiplatelet Resistance

The evaluation of antiplatelet resistance is characterised by considerable variation, employing an array of different platelet function tests and utilising the downstream metabolites of arachidonic acid breakdown, such as serum thromboxane B2 (TxB2) or its urinary metabolite, 11-dehydro-TxB2. These metabolites serve to reflect the impact of aspirin on platelet function.

#### 3.2.1. Light Transmission Aggregometry

The preparation of platelet-rich plasma (PRP) involves centrifuging a 5 ml anticoagulated blood sample at 150g for 10 minutes at room temperature. Subsequently, the PRP is calibrated to a platelet count ranging from 150,000 to 300,000 *µ*l. Light transmission aggregometry is then employed to assay the samples, necessitating the addition of 0.05 ml of arachidonic acid. However, the process can also be undertaken without the adjustment of the platelet count, and alternatives such as type I collagen and ADP may be used in lieu of arachidonic acid [11]. The degree of aggregation is plotted as a function of time and represented as the total percentage of aggregation at the five-minute mark [14, 24].

#### 3.2.2. Impedance Platelet Aggregometry

Multiple Electrode Aggregometry (MEA) assesses platelet aggregation through the continual monitoring of alterations in electrical impedance, attributable to the activation and subsequent adherence of platelets to metal sensor electrodes across 3–5 distinct channels [12, 15, 16, 19, 21–23, 29]. Each channel utilises a whole blood sample, with arachidonic acid added to evaluate the impact of aspirin (ASPItest), ADP for assessing the effect of P2Y12 platelet inhibitors (ADPtest), or thrombin receptor agonist peptide for measuring the impact of glycoprotein IIb/IIIa inhibitors (TRAPtest) [12, 16, 19, 23]. Additionally, collagen can be employed as an alternative to arachidonic acid for ascertaining the effect of aspirin [22]. The resultant aggregation data are presented as an arbitrary area under the curve (AUC) or expressed as an aggregation unit over time (AU x min).

#### 3.2.3. Platelet Function Assay (PFA)

The PFA-100 (Dade Behring, Germany) is a commercially accessible point-of-care platelet functionality assay that gauges platelet activation under considerable shear stress by aspirating whole blood through cartridges coated with either collagen/epinephrine (CEPI) or collagen/ADP (CADP) [11, 25]. The evaluation is documented as Aperture Closure Time (CT), denoting the duration required for ensuing platelet activation to occlude the apertures within the CEPI and CADP cartridges.

#### 3.2.4. VerifyNow Assay

The VerifyNow system (Accumetrics, San Diego, CA, USA) represents a cartridge-based rapid assay apparatus assessing aspirin impact on platelet reactivity through the VerifyNow Aspirin Test, utilising arachidonic acid as an agonist. Conversely, the VerifyNow P2Y12 Test gauges the direct inhibition of clopidogrel on P2Y12 receptors [13, 26]. Aspirin test findings are represented as Aspirin Reaction Units (ARUs), while P2Y12 test results are documented as P2Y12 Reaction Units (PRUs).

#### 3.2.5. Thromboelastogram (TEG)

Heparinised whole blood is used in the TEG assay (Haemoscope Corp, Niles, IL, USA) to evaluate the platelet function in terms of clot maximum amplitude with added arachidonic acid (MA_AA_) or without a platelet agonist (MA_0_), which is compared with kaolin-activated TEG assay (MA_KH_) to derive percent of platelet aggregation using the formula: %MA_AA_ = [(MA_AA_ − MA_0_)/(MA_KH_ − MA_0_)] × 100% [22]. The result is reported as a percent aggregation of platelets.

#### 3.2.6. Whole-Blood Flow Cytometry

Antiplatelet resistance can be quantified via a process involving blood incubation with or devoid of arachidonic acid (1.0 mmol/L) for a duration of two minutes, followed by the addition of radiolabeled antibodies targeting CD41a or CD62P receptors on platelets. Postfixation of the samples with 1% paraformaldehyde, the ensuing analysis is conducted using a fluorescent cell sorter (Becton-Dickinson FACScan; BD Immunocytometry Systems, San Jose, CA, USA) [22]. The outcome is articulated as the percentage augmentation in the expression of the CD62P receptor following activation.

#### 3.2.7. Thromboxane B2 (TxB2)

Urinary 11-dehydro-TxB2, the excreted form of TxB2, can typically be quantified using enzyme immunoassay kits (Cayman Chemical, MI, USA), with results being normalised to the urinary creatinine concentration [11, 14, 24]. Serum TxB2 levels, indicative of cyclooxygenase-2-dependent thromboxane biosynthesis, can be gauged in the plasma obtained from whole blood cultured at 37°C for a 24-hour duration and subsequently centrifuged at 700 × g for 15 minutes [11]. Serum TxB2 levels can alternatively be measured employing immunoassay or radioimmunoassay methods [15, 28]. Another approach involves using centrifuged plasma to quantify serum 11-dehydro-TxB2 levels utilising an enzyme immunoassay kit (Assay Designs Inc., Ann Arbor, MI, USA) [22].

### 3.3. Definition of Antiplatelet Resistance

The definition of antiplatelet resistance in the current literature is not uniform, with its interpretation varying significantly across different assessment methods (Table 2). According to light transmission aggregometry, platelet aggregation of ≥20% with arachidonic acid is considered indicative of aspirin resistance, while some studies set the threshold at an aggregation of >30% [17, 18, 24]. Impedance aggregometry defines aspirin resistance as an AUC ≥30 units or AUCASPI >300 units (APSItest value >75 percentile) [15, 16, 21–23, 29–31].

In the context of the VerifyNow system, values of aspirin reaction units (ARUs) >550 and P2Y12 reaction units (PRUs) >230 are interpreted as aspirin and clopidogrel resistance, respectively, Nevertheless, the PRU cutoff point for clopidogrel resistance could be as low as 188 in certain cases [13, 26, 27]. Aspirin resistance is defined as a collagen and/or epinephrine (CEPI) closure time of <193 seconds in the PFA-100 system [25].

Furthermore, resistance to aspirin is characterised by an inhibition of serum TxB2 of less than 90%, an increase in serum 11-dehydro-TxB2 of >25% from baseline, and urinary 11-dehydro-TxB2 levels exceeding 67.9 ng/mmol of creatinine [14, 15, 22]. Aspirin resistance is also defined by platelet aggregation of >50% in TEG and a 25% increase of the CD62P receptor expression following simulation in whole-blood flow cytometry [22, 32, 33].

### 3.4. Antiplatelets Used in the Studies

All investigations incorporated aspirin as the principal antiplatelet treatment, with clopidogrel supplementing aspirin to form a dual antiplatelet therapy in several instances [15, 24, 26, 30, 31]. Though clopidogrel is invariably administered at a dosage of 75 mg, the dosage of aspirin displays variability in the range of 80–325 mg, with 100 mg being the most frequently prescribed dosage. One study employed a postoperative loading dose of intravenous aspirin of 500 mg [18].

### 3.5. Incidence of Antiplatelet Resistance

The recorded prevalence of overall aspirin resistance spanned from 11 to 51.5%, whereas the incidence of resistance to clopidogrel was reported to be 22% [13, 16–18, 21–25, 30]. Among patients receiving dual antiplatelet therapy, 12.6% exhibited resistance to aspirin and clopidogrel; however, this proportion declined to 10.6% after a 30-day treatment regimen [13]. Preoperative aspirin resistance was observed in 13–29% of cases [16–18].

As for TxB2 measurements, inhibition exceeding 90% was not obtained until five days after surgery, and merely 34% of patients had platelet inhibition by this point [14, 15]. Inadequate inhibition of TxB2 was observed with a dosage of 100 mg aspirin, but this was not the case when the dosage was increased to 325 mg [11].

In patients who had demonstrated aspirin resistance perioperatively, this resistance had dissipated in all instances when retested at 6-month and 12-month follow-ups [17, 18, 24].

### 3.6. Effect of Cardiopulmonary Bypass (CPB)

The effect of CPB on aspirin resistance remains ambiguous within the literature. Platelet aggregation and thromboxane exhibit notable suppression subsequent to off-pump CABG, whereas such substantial inhibition is not observed after on-pump CABG [28]. A separate investigation delineates CPB duration as an independent predictor of aspirin nonresponse [23]. Contrarily, several studies have asserted that CPB does not significantly influence aspirin resistance [17, 18].

### 3.7. Outcomes

#### 3.7.1. Vein Graft Occlusion

Antiplatelet resistance serves as a predictive factor for graft occlusion [13]. Aspirin resistance, when concomitant with compromised vein graft endothelial integrity, precipitates graft thrombosis and failure within a few days post-CABG [22]. Furthermore, late occlusion of vein grafts exhibits a thirteen-fold increase in risk (expressed as an odds ratio) in the presence of aspirin resistance [25]. Dual antiplatelet therapy represents a potent predictor of vein graft patency and is associated with a decreased incidence of vein graft occlusion [13].

#### 3.7.2. Mortality, Myocardial Infarction (MI), and Stroke

There was no significant difference observed in mortality rates, MI, or stroke incidents between patients demonstrating aspirin resistance and those without it. One study demonstrated this during a 6-month follow-up [30, 31]. A further two studies demonstrated it during the 12-month follow-up periods [16, 23]. However, it is noteworthy to mention that all patients who died during the 12-month follow-up duration in the other two studies had previously displayed signs of aspirin resistance [17, 18].

The addition of clopidogrel to the aspirin did not result in a decrease in adverse outcomes or an increase in bleeding incidents, except in a specific population with younger obese patients (age <65 years, BMI >30), where the incidence of adverse events was lower compared to aspirin monotherapy [30]. This is because patients with a BMI >30, who are aspirin resistant, have worse adverse outcomes compared to those without it [31]. Moreover, in patients resistant to clopidogrel and undergoing off-pump CABG, a high residual platelet reactivity is linked with elevated mortality rates, MI, and target vessel revascularisation [26].

#### 3.7.3. Postoperative Immediate Blood Loss

The volume of postoperative blood loss 12 hours after surgery was observed to be higher in patients sensitive to preoperative aspirin in comparison to those displaying preoperative aspirin resistance, with mean volumes amounting to 555 ml and 406 ml, respectively [27]. Although the chest drain output was comparable within the first hour following surgery, a greater blood loss was recorded in the aspirin-sensitive group at both the 6-hour and 12-hour marks. Furthermore, these patients exhibited a higher risk of requiring blood transfusion in the postoperative period [19].

## 4. Discussion

True resistance to the inhibition of thromboxane A2—essentially, resistance to the biochemical effects of aspirin—is an infrequent phenomenon. Conversely, the incidence of thrombotic events and suboptimal clinical outcomes in spite of aspirin usage in patients could be attributable to an array of mechanisms extending beyond mere inhibition of the COX-1 enzyme [34]. Consequently, the terminology “antiplatelet resistance” lacks a universal definition in the literature. Nevertheless, antiplatelet resistance, when detected with in vitro platelet assays, is associated with adverse clinical outcomes in patients receiving antiplatelet therapy [35–38].

Reports of antiplatelet resistance in patients undergoing cardiac surgery vary due to differing cutoff values for measurements, even when using identical assessment methodologies. Moreover, these inconsistencies are amplified when employing disparate measurement methods. For instance, one study identified aspirin resistance with light aggregometry when platelet aggregation was ≥20%, while others set the threshold at >30% [17, 18, 24].

These variant assessment methods yield differing results in determining antiplatelet resistance, thus compromising the comparability between studies [11]. A patient labelled as resistant to antiplatelets in one study might not receive the same categorisation in another study utilising a different assessment method. Furthermore, the range of aspirin dosages used across individual studies might influence the manifestation of aspirin resistance.

Moreover, despite the predominant focus on aspirin resistance in studies assessing antiplatelet resistance, there is a lack of information concerning resistance to other antiplatelet agents, such as clopidogrel. Although many assessment methods provide the capability to test clopidogrel resistance by using ADP as an alternative to arachidonic acid as a substrate, this capability is not widely employed. Although clopidogrel is commonly used in numerous studies, only Mannacio et al. reported the incidence of clopidogrel resistance [13, 21, 26, 29, 30].

Prior studies involving noncardiac surgery cohorts have shown a correlation between antiplatelet resistance and increased cardiovascular thrombotic events and mortality rates [37, 39, 40]. While a number of randomised controlled trials and observational studies did not report a significant difference in adverse outcomes in cardiac surgery patients, including mortality, stroke, and myocardial infarction, these studies did not examine graft patency or patient symptoms [16, 23, 30, 31]. The absence of differences in adverse outcomes may be due to the potentially transient nature of aspirin resistance [17, 18, 24]. Notably, aspirin resistance is linked to decreased blood loss in the immediate postoperative period, which could be interpreted as a prothrombotic feature when compared with the aspirin-sensitive population [27].

Better clinical outcomes were observed in a subset of younger (<65 years) and obese aspirin-resistant patients when dual antiplatelet therapy with clopidogrel was administered [30]. Additionally, all patients who died during the follow-up period were initially identified as having perioperative aspirin resistance [17, 18]. Youn et al. documented worse outcomes in patients undergoing cardiac surgery with clopidogrel resistance [26]. The assessment of this patient cohort could be further augmented with follow-up coronary angiograms and/or computed tomography coronary angiography.

Antiplatelet resistance is not limited to CABG surgery alone. This resistance contributes to poor clinical outcomes in cardiovascular disease, whether managed medically or through percutaneous coronary interventions, as well as in cerebrovascular diseases, including stroke and neuro-interventional procedures [37, 41–43]. A comprehensive meta-analysis including 2,930 patients demonstrated that antiplatelet resistance is associated with a significantly higher incidence of cardiovascular events (odds ratio (OR): 3.85; 95% confidence interval (CI): 3.08–4.80) and mortality (OR: 5.99; 95% CI: 2.28–15.72) [37]. Consequently, individuals with antiplatelet resistance are at an elevated risk of long-term morbidity and mortality.

## 5. Limitations

This systematic review is subject to certain limitations. Predominantly, the studies incorporated in this review are observational cohort studies as opposed to randomised controlled trials. Due to the deployment of diverse assessment methods for antiplatelet resistance and their varied results, conducting a meta-analysis is impracticable. Further compounding this issue is the absence of a uniform definition for antiplatelet resistance across different methods. Moreover, the results could potentially be swayed by various surgical techniques and vein graft handling and management, whose precise effects remain largely obscure, alongside the influence of antiplatelet resistance.

Future investigations with adequately powered randomised controlled trials are required to explore the outcomes of antiplatelet resistance. This should encompass the resistance of other antiplatelet agents, thus moving beyond a narrow focus on aspirin as the main antiplatelet agent. To better understand the clinical significance of resistance to antiplatelet medication, more extensive imaging studies need to be undertaken to take into account the quality of the grafted coronary artery.

## 6. Conclusion

The existing literature lacks a consistent definition of antiplatelet resistance. The methods used to evaluate antiplatelet resistance vary significantly, leading to diverse and noninterchangeable results. Although the focus of these studies predominantly rests on aspirin resistance, information regarding other antiplatelets like clopidogrel and ticagrelor remains scarce. Antiplatelet resistance in patients undergoing CABG surgery is correlated with an elevated rate of vein graft occlusion. While aspirin resistance has a conflicting impact on overall adverse outcomes, the presence of clopidogrel resistance is associated with worsened outcomes in CABG patients.

## Figures and Tables

**Table 1:** Characteristics of the included studies.

Author	Year	Study design	No. of patients	Antiplatelet	Measurements	Conclusion
Bednar et al.	2009	Prospective cohort	40	Aspirin	Platelet aggregation, 11-dehydrothromboxane B2	Aspirin does not inhibit platelet aggregation sufficiently in early postoperative days
Bednar et al.	2012	Prospective cohort	30	Aspirin + clopidogrel	Platelet aggregation, 11-dehydrothromboxane B2	Aspirin-induced inhibition of thromboxane B2 production and platelet aggregation is impaired in the early days of the postoperative period
Bollinger et al.	2016	Prospective cohort	304	Aspirin	Platelet aggregation	Reduced aspirin responsiveness is not associated with major adverse events after CABG
Brambilla et al.	2010	Randomised controlled trial	56	Aspirin	Platelet aggregation, 11-dehydrothromboxane B2	Incidence of antiplatelet resistance is lower with a higher dosage of aspirin
Gasparovic et al.	2014	Randomised controlled trial	219	Aspirin	Platelet aggregation	Addition of clopidogrel to aspirin-resistant patients does not reduce adverse outcomes nor increase bleeding
Hiyasat et al.	2014	Prospective cohort	100	Aspirin	Platelet aggregation	Aspirin resistance occurs in a large population of patients after CABG and is associated with worse outcomes
Kempfert et al.	2009	Prospective cohort	59	Aspirin	Platelet aggregation	Aspirin resistance is a transient phenomenon in a large population of patients undergoing CABG
Mannacio et al.	2012	Randomised controlled trial	300	Aspirin	Platelet function assay	Aspirin resistance is a predictor of vein graft patency, and dual antiplatelet therapy improves it
Nicola et al.	2019	Prospective cohort	250	Aspirin	Platelet aggregation	Aspirin effects slightly increase blood loss and the requirement of allogeneic blood transfusion
Petricevic et al.	2011	Prospective cohort	99	Aspirin	Platelet aggregation	Postoperative aspirin administration does not sufficiently inhibit platelet aggregation in CABG patients
Petricevic et al.	2013	Prospective cohort	131	Aspirin + clopidogrel	Platelet aggregation	Dose adjustment or dual antiplatelet therapy should be considered in aspirin-resistant patients to reduce adverse events
Petricevic et al.	2015	Randomised controlled trial	325	Aspirin	Platelet aggregation	An exploratory analysis found no significant impact of aspirin resistance on outcomes in patients undergoing coronary artery bypass grafting
Poston et al.	2006	Prospective cohort	225	Aspirin	Platelet aggregation, 11-dehydrothromboxane B2, whole-blood flow cytometry, thromboelastogram	Aspirin resistance and compromised endothelial integrity lead to vein graft failure within a few days after off-pump CABG surgery
Wand et al.	2017	Prospective cohort	400	Aspirin	Platelet aggregation	High incidence of perioperative aspirin nonresponse rate does not increase 1-year cardiovascular adverse event rate
Wang et al.	2012	Prospective cohort	333	Aspirin + clopidogrel	Platelet aggregation, 11-dehydrothromboxane B2	Aspirin resistance is a transient phenomenon in the early postoperative period
Yilmaz et al.	2005	Prospective cohort	28	Aspirin	Platelet function assay	Aspirin resistance is highly prevalent in patients with late occlusion of vein grafts
Youn et al.	2014	Prospective cohort	859	Aspirin + clopidogrel	Platelet function assay	High residual platelet activity after clopidogrel administration is associated with a higher 1-year adverse event rate
Willemsen et al.	2021	Prospective cohort	128	Aspirin	Platelet function assay	Aspirin-sensitive patients have more 12-hour blood loss after CABG
Zimmermann et al.	2005	Prospective cohort	29	Aspirin	Platelet aggregation	The antiplatelet effect of aspirin is largely impaired after on-pump, but not off-pump CABG

**Table 2:** Summary of antiplatelet resistance measurement methods.

Measurements	No. of studies	No. of patients	Antiplatelet resistance
Platelet aggregation	15	2,600	If aggregation ≥20–30% (light aggregometry), AUC ≥30 units or AUCASPI >300 units (impedance aggregometry)
Platelet function assay	4	1,315	If ARU >550, PRU >230 (VerifyNow), CEPI <193 seconds (PFA system)
11-Dehydrothromboxane B2	5	684	If 11-dehydro-TxB2 >67.9 ng/mmol of creatinine
Whole-blood flow cytometry	1	225	If 25% increase of the CD62P receptor expression
Thromboelastogram	1	225	If platelet aggregation >50%

## Data Availability

Data are openly available in a public repository that issues datasets with DOIs.

## Abbreviations

ACS: Acute coronary syndrome
ADP: Adenosine diphosphate
ARU: Aspirin reaction unit
AUC: Area under the curve
BMI: Body mass index
CABG: Coronary artery bypass grafting
CADP: Collagen/ADP-coated apertured cartridges
CEPI: Collagen/epinephrine-coated apertured cartridges
CPB: Cardiopulmonary bypass
MA: Maximum amplitude
MEA: Multiple electrode aggregometry
MI: Myocardial infarction
NOS: Newcastle-Ottawa Scale
PFA: Platelet function assay
PRISMA: Preferred Reporting Items for Systematic Reviews and Meta-Analyses
PRP: Platelet-rich plasma
PRU: P2Y12 reaction unit
TEG: Thromboelastogram
TxA2: Thromboxane A2
TxB2: Thromboxane B2.

## Supplementary Materials

Supplementary Materials Supplementary Table 1: quality assessment of the included cohort studies using the Newcastle-Ottawa Scale (NOS). Supplementary Figure 1: risk of bias summary of the included randomised controlled trials.
