# Supplementary material for: Antiplatelet Resistance in Coronary Artery Bypass Grafting: A Systematic Review
Source: Surg Res Pract. 2024 Jun 15;2024:1807241. doi: 10.1155/2024/1807241 (PMC11193597; doi:10.1155/2024/1807241)
Supplement: Supplementary Materials — Supplementary Table 1: quality assessment of the included cohort studies using the Newcastle-Ottawa Scale (NOS). Supplementary Figure 1: risk of bias summary of the included randomised controlled trials. [file 1807241.f1.docx]

# Supplementary Table

**Supplementary Table 1: Quality assessment of the included cohort studies using the Newcastle-Ottawa Scale (NOS)**

| Study ID | Selection of Study Groups  (0-4) | | | | Comparability  (0-2) | Assessment of Outcome  (0-3) | | | Total Score  (0-9) |
| --- | --- | --- | --- | --- | --- | --- | --- | --- | --- |
|  | Representativeness of the exposed cohort | Selection of the non-exposed cohort | Ascertainment of exposure | Demonstration of the outcome is not present at the beginning | Comparability of cohorts on the basis of the design or analysis | Assessment of outcome | Enough follow-up length for outcomes to occur | Adequacy of follow-up of cohorts |  |
| Bednar 2009 | 1 | 0 | 1 | 1 | 0 | 1 | 1 | 1 | 6 |
| Bednar 2012 | 1 | 0 | 1 | 1 | 0 | 1 | 1 | 1 | 6 |
| Bollinger 2016 | 1 | 0 | 1 | 1 | 0 | 1 | 1 | 1 | 6 |
| Hiyasat 2014 | 1 | 0 | 1 | 1 | 0 | 1 | 1 | 1 | 6 |
| Kempfert 2009 | 1 | 0 | 1 | 1 | 0 | 1 | 1 | 1 | 6 |
| Nicola 2019 | 1 | 0 | 1 | 1 | 0 | 1 | 1 | 1 | 6 |
| Petricevic 2011 | 1 | 1 | 1 | 1 | 2 | 1 | 1 | 1 | 9 |
| Petricevic 2013 | 1 | 1 | 1 | 1 | 2 | 1 | 1 | 1 | 9 |
| Poston 2006 | 1 | 0 | 1 | 1 | 0 | 1 | 1 | 1 | 6 |
| Wand 2017 | 1 | 0 | 1 | 1 | 0 | 1 | 1 | 1 | 6 |
| Wang 2012 | 1 | 0 | 1 | 1 | 0 | 1 | 1 | 1 | 6 |
| Yilmaz 2005 | 1 | 0 | 1 | 1 | 0 | 1 | 1 | 1 | 6 |
| Youn 2014 | 1 | 0 | 1 | 1 | 0 | 1 | 1 | 1 | 6 |
| Zimmermann 2005 | 1 | 0 | 1 | 1 | 0 | 1 | 1 | 1 | 6 |

Good quality (7-9 score), Fair quality (4-6 score), Poor quality (0-3 score)

#

# Supplementary Figure


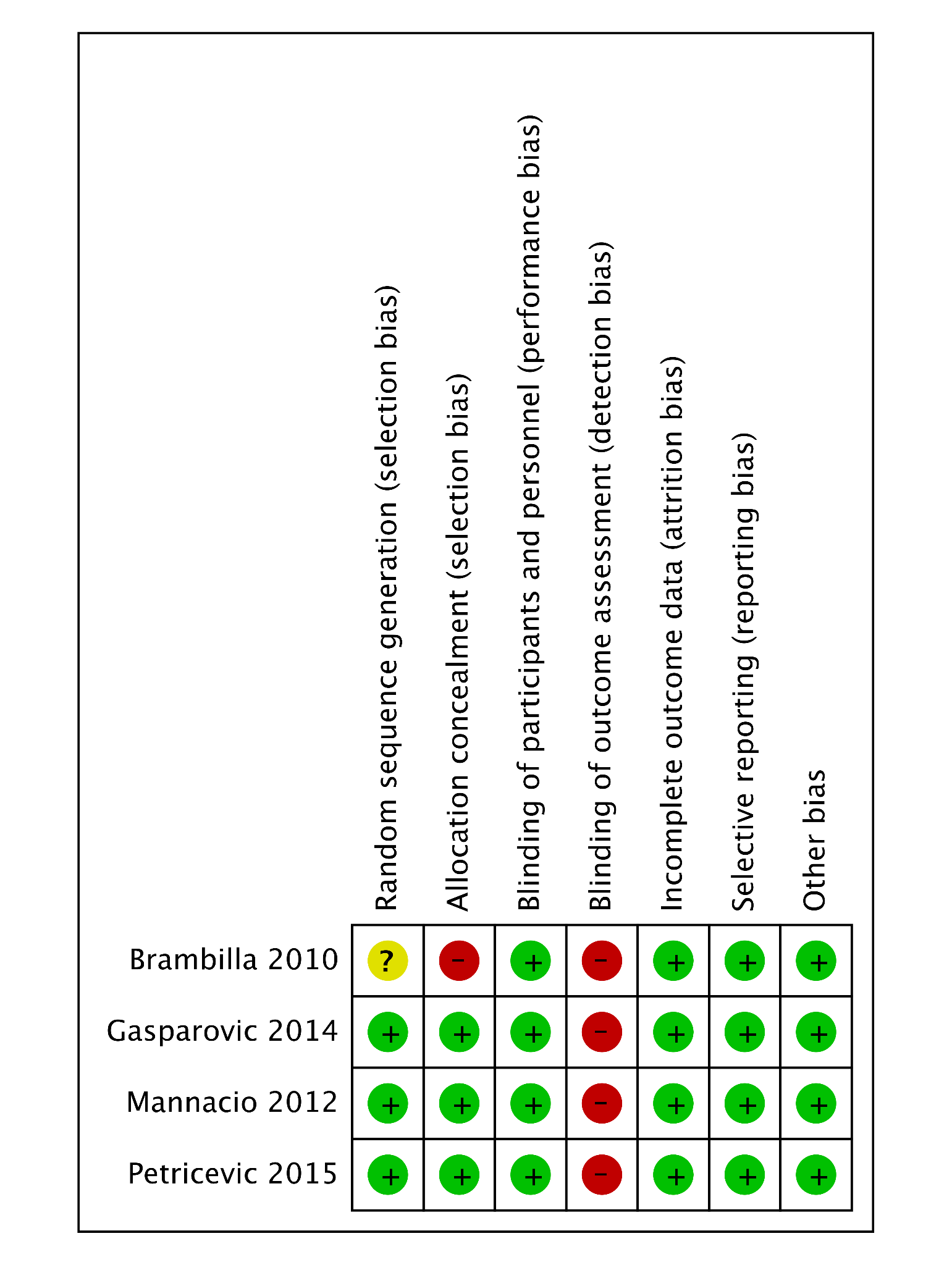


Supplementary Figure 1: Risk of bias summary of the included randomised controlled trials
